# Multidimensional assessment of in-host fitness costs of echinocandin resistance in the opportunistic fungal pathogen *Candida glabrata* reveals the niche-specific requirement for *FKS1* and *FKS2* during infection and gut colonization

**DOI:** 10.1128/aac.01801-25

**Published:** 2026-03-23

**Authors:** Amir Arastehfar, Farnaz Daneshnia, Hrant Hovhannisyan, Nathaly Cabrera, Sebastian Jusuf, Mostafa Salehi, Michael K. Mansour, Toni Gabaldón, Erika Shor, David S. Perlin

**Affiliations:** 1 Division of Infectious Diseases, Massachusetts General Hospital 2348, Boston, Massachusetts, USA; 2 Department of Medicine, Harvard Medical School 205260 , Boston, Massachusetts, USA; 3 University Hospital Wuerzburg, Medical Hospital II, Würzburg, Germany; 4 Institute of Biodiversity and Ecosystem Dynamics (IBED), University of Amsterdam 1234, Amsterdam, the Netherlands; 5 Life Sciences Programme, Supercomputing Center (BSC-CNS) 132144 , Barcelona, Spain; 6 Institute for Research in Biomedicine (IRB Barcelona), The Barcelona Institute of Science and Technology 518635, Barcelona, Spain; 7 Center for Discovery and Innovation, Hackensack Meridian Health 721734, Nutley, New Jersey, USA; 8 Department Industrial Engineering Faculty of K.N, Toosi University of Technology 108871, Tehran, Iran; 9 Catalan Institution for Research and Advanced Studies 117370, Barcelona, Spain; 10 Centro de Investigación Biomédica en Red de Enfermedades Infecciosas (CIBERINFEC) 637284 , Barcelona, Spain; 11 Georgetown University Lombardi Comprehensive Cancer Center 66634, Washington, DC, USA; 12 Department of Medical Sciences, Hackensack Meridian School of Medicine 576909, Nutley, New Jersey, USA; University of Iowa, Iowa City, Iowa, USA

**Keywords:** *Candida glabrata*, echinocandin, echinocandin resistance, fitness cost, intracellular replication, neutrophil, cell wall, gut colonization, systemic infection

## Abstract

Host stresses are often considered a major barrier against the emergence of echinocandin resistance (ECR) in prominent infecting organisms like *Candida albicans* due to fitness defects. Yet, ECR strains of *C. glabrata* carrying diverse amino acid changes in Fks1 and Fks2 are increasingly reported as breakthrough infections. Nonetheless, the impact of equivalent mutations in different *FKS* alleles on fitness has not been systematically studied. Herein, we employed a diverse array of *ex vivo* and *in vivo* models to address these questions among clinically relevant ECR mutants. All ECR mutants retained fitness during interaction with THP1 macrophages and neutrophils. Whereas a strain with a Fks2^F659del^ or *fks2Δ* showed fitness defects during interaction with macrophages and neutrophils. Fks2^F659del^ showed a unique susceptibility to numerous stresses, especially the combination of alternative carbon sources, low pH, and H_2_O_2_. Consistent with failure in mounting adaptive oxidative stress response during exposure to H_2_O_2_, transcriptomic analysis of intracellular Fks2^F659del^ highlighted the dysregulation of oxidative stress response genes, whereas intracellular *fks2Δ* showed hallmarks of metabolic dysregulation. Intriguingly, the Fks2^F659del^ mutant was outcompeted by wild type and Fks2^F659V^ in *in vivo* gut colonization and systemic infection models. Importantly, whereas both *FKS1* and *FKS2* were required to establish gut colonization, only *FKS2* was required for systemic infection. Therefore, our study supports the notion that the prevalence of ECR mutants among *C. glabrata* strains is likely driven by its ability to retain fitness across diverse niches. Furthermore, we identified that the essentiality of *FKS1* and *FKS2* is similarly dictated by niche-specific requirements.

## INTRODUCTION

The increasing rate of drug-resistant microbes stands as one of the major public health threats facing modern medicine (1). The severity of the emerging threat of drug resistance is more profound for fungal pathogens, since only a limited number of antifungal drugs are available to treat invasive fungal infections (2, 3). The extensive use of azoles and the parallel increase in the number of azole-resistant fungal pathogens, coupled with the nephrotoxicity of amphotericin B (AMB), have elevated echinocandins as the frontline antifungal drugs to treat a variety of candidiasis, including bloodstream *Candida* infections, that is, candidemia (4). Although less commonly than azole resistance, the number of echinocandin-resistant (ECR) isolates of various *Candida* species, especially *Candida glabrata (Nakaseomyces glabratus*), has been increasing in numerous clinical centers (5–11). Echinocandins are fungicidal drugs that target the catalytic subunit of 1,3-β-D-glucan synthase, encoded by *FKS1* and *FKS2* genes, which produces one of the major structural components of the cell wall. Accordingly, ECR *C. glabrata* isolates typically harbor mutations in short regions of *FKS1* and *FKS2*, known as hotspot 1 (HS1) and 2 (HS2), which reduce the sensitivity of glucan synthase to echinocandin drugs presumably by altering echinocandin binding (2, 3).

Since 1,3-β-D-glucan constitutes an integral component of the cell wall and *FKS* mutations are shown to decrease 1,3-β-D-glucan synthase catalytic activity (12) and its biosynthetic capacity, ECR mutants carry a fitness cost (13, 14). Consistently, ECR *C. albicans* isolates carrying *FKS1* mutations have severe growth and filamentation defects and consequently are hypovirulent in the context of *in vivo* infection models (13, 14). Such severe fitness costs potentially underlie the low epidemiological prevalence of ECR *C. albicans* isolates in the clinic (13–15) and have been used as a proxy to hypothesize that restriction of echinocandin use may be pivotal in curtailing the prevalence of ECR *C. albicans* isolates in the clinic (13, 14).

Yet, the higher rate of ECR *C. glabrata* isolates in the clinic is in sharp contrast with the *C. albicans* observations and indicates that ECR *C. glabrata* isolates carrying *FKS* mutations may have a more subtle fitness cost (5–10). Consistent with this notion, experimentally evolved ECR *C. glabrata* isolates show no significant differences in fitness in rich medium (16), and their resistance is rarely lost after removal of drug exposure (17). Additionally, it was shown that fitness-deficient fluconazole-resistant *C. glabrata* isolates can be rescued by introducing HS1 mutations in *FKS1*/*2*, the degree of which is dictated by the mutation type and allele (18). Therefore, *FKS* mutations can be tolerated by *C. glabrata,* potentially by bestowing protection against host-related stresses (17). Nonetheless, epidemiological studies have noted that (i) some mutations are more prevalent than others and (ii) more than 2/3 of the ECR *C. glabrata* isolates carry *FKS2* mutations (5, 7, 8, 10, 19). For instance, ECR isolates carrying amino acid substitutions Fks1-S629P and Fks2-S663P are highly prevalent, whereas some others, including Fks1-R631G and Fks2-R665G, are less prevalent (5, 7, 8, 10, 19). Additionally, noting that multiple factors impact the survival of candidemia patients, anecdotal evidence suggests that patients infected with ECR isolates carrying Fks2-S663P have a poorer prognosis (7, 10) than those infected with Fks2-F659del (10). Therefore, a better understanding of the impact of diverse *FKS*-mediated ECR mutations and fitness during systemic infection may have broader clinical implications in some settings to improve clinical outcomes. Nonetheless, the impact of ECR-causing mutations on fitness in *C. glabrata* is poorly understood. Moreover, unlike *C. albicans*, both *FKS1* and *FKS2* seemed to be operational in *C. glabrata*, as supported by both genes harboring acquired mutations in ECR isolates. Nonetheless, *FKS1* and *FKS2* are differentially regulated (20, 21), and their roles in the context of interaction with phagocytes and during gastrointestinal (GI)-tract colonization and systemic infection have not been characterized.

To address these knowledge gaps, we used isogenic *C. glabrata* isolates carrying equivalent mutations in the HS1 of *FKS1* and *FKS2,* as well as those harboring distinct mutations at the same residue, and assessed their fitness using an extensive array of *in vitro*, *ex vivo*, GI-tract colonization, and systemic infection models. We show that, unlike most ECR point mutants in *FKS2*, those carrying Fks2^F659del^ are particularly unfit during interaction with macrophages and neutrophils, and we observed the same defect for clinical isolates carrying Fks2^F659del^. Similarly, we found that *fks2Δ* mutants show severe fitness defects during interaction with macrophages and neutrophils, whereas *FKS1* was dispensable during interaction with these phagocytes. As shown by our *in vitro* and RNAseq analysis, dysregulation in mounting an adaptive oxidative stress response (OSR) would appear to underlie the intracellular fitness defect of Fks2^F659del^, whereas metabolic dysregulation at the intracellular stage is a major hallmark of *fks2Δ* mutants. Finally, while *FKS2* was found to be essential to establish gut colonization and systemic infections, *FKS1* was only required for effective GI-tract colonization. Interestingly, Fks2^F659del^ mutants were unfit during both GI-tract colonization and systemic infection, whereas point mutant counterparts carrying Fks2^F659V^ only showed fitness cost in the kidney and competed effectively with the susceptible wild type (WT) in the GI-tract and spleen. Overall, our multidimensional fitness assessment of ECR *C. glabrata* isolates offers clues on their epidemiological prevalence, potentially linking the observed notable prevalence of *C. glabrata* ECR isolates to the subtle fitness costs in various host-related ecological niches. Finally, our study provides a unique perspective on the implications of *FKS1* and *FKS2* during GI-tract colonization and systemic infection.

## RESULTS

### Intracellular replication rate varies depending on the nature of the *FKS* amino acid substitution

We previously showed that the intracellular replication (IR) rates of clinical and isogenic ECR *C. glabrata* isolates were comparable to their susceptible counterparts (18). Moreover, we noted that IR defects observed in FLZR *C. glabrata* isolates were rescued by *FKS* mutations, with rescue levels being higher for *FKS2* mutants as compared to *FKS1* mutants (18). In that study, isogenic ECR mutants carrying different mutations at the same residue (Fks2^F659del^ vs Fks2^F659V^) or equivalent mutations in HS1 of *FKS1* (S629P and R631G) and *FKS2* (S663P and R665G) compared to their susceptible WT were not investigated. The logic behind the selection of these mutations was either their prevalence in the clinic and/or their potential fitness as inferred from anecdotal studies. For instance, ECR mutants carrying Fks1^S629P^ and Fks2^S663P^ are more predominant as compared to counterparts carrying Fks1^R631G^ and Fks2^R665G^ (5, 7, 8, 10, 19), whereas as opposed to patients infected with Fks2^S663P^ (7, 10), those infected with ECR isolates carrying Fks2^F659del^ have a better prognosis (validation of which requires extensive clinical studies) (10). Moreover, those amino acid changes occur in both Fks1 and Fks2, both of which seem to be required for β-glucan synthesis.

**Fig 1:**
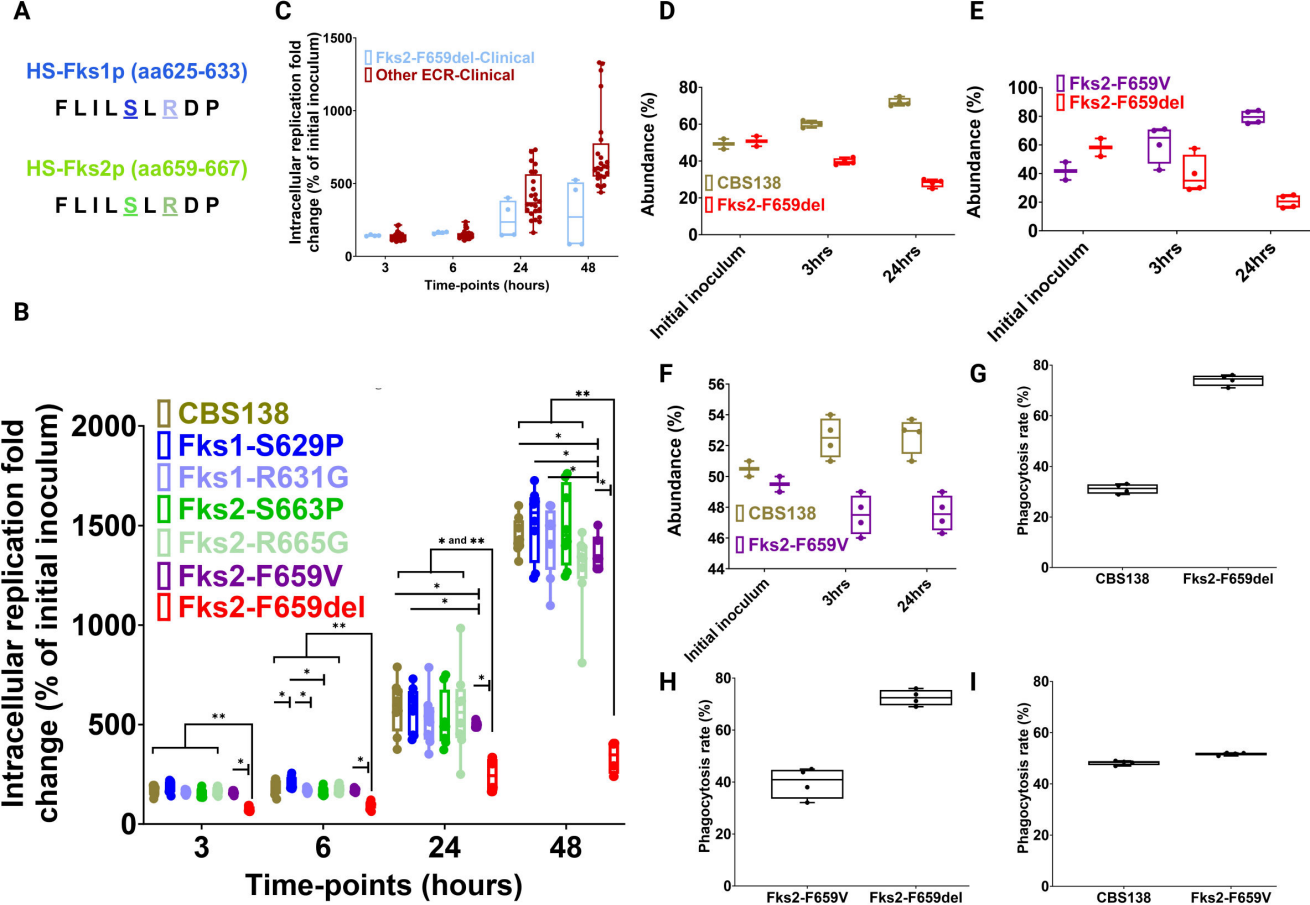
Schematic representation of various mutations investigated in this study (note that aa refers to amino acid) (**A**). Except for Fks2^F659del^, the rest of the echinocandin-resistant colonies showed comparable replication rate relative to susceptible wild type (CBS138) within THP1 macrophages (**B**). Similarly, clinical ECR strains Fks2^F659del^ mutants (*n* = 2) showed a severe intracellular replication rates within THP1 macrophages compared to the rest of the ECR isolates (*n* = 11) carrying other mutations (**C**). Intracellular competition of Fks2^F659del^ vs wild type (**D**), Fks2^F659del^ vs Fks2^F659V^ (**E**), Fks2^F659V^ vs wild type (**F**), phagocytosis of Fks2^F659del^ vs wild-type competition (**G**), Fks2^F659del^ vs Fks2^F659V^ competition (**H**), and Fks2^F659V^ vs wild type (**I**). Each data point reflects an individual biological replicate in all figures. The Wilcoxon test was used for analysis. *, **, ***, and **** refer to *P* values <0.05 and >0.01, <0.01, <0.005, and <0.0005, respectively. THP1: Tohoku Hospital Pediatrics-1.

To better assess the contributions of the mutations, isogenic ECR isolates carrying equivalent mutations (S629P vs S663P and R631G vs R665G) were generated by CRISPR-Cas9, and ECR mutants carrying different mutations at the same residue (F659del vs F659V) were generated by exposure to caspofungin (Fig. 1A). All isogenic echinocandin-resistant mutants (iECR) shared the same *C. glabrata* CBS138 background. The genotypes and phenotypes of all mutants were confirmed by Sanger sequencing the HS1 and HS2 of *FKS1* and *FKS2* and by echinocandin susceptibility testing. Notably, whole-genome sequencing revealed that ECR mutants evolved through caspofungin exposure did not carry additional mutations throughout the genome (see Materials and Methods section and Table S1).

To assess the IR rates of the iECR and susceptible strains, THP1 macrophages were infected at a multiplicity of infection (MOI) of 1/1. The macrophages were washed with PBS to remove the non-adherent yeast cells, fresh RPMI at 3 h post-infection (pi) was added, and the macrophages were lysed, and the lysate was plated on agar plates at 3, 6, 24, and 48 h pi. The IR rate was calculated by dividing the colony-forming units (CFUs) obtained at each time-point by the CFU of the initial inoculum. As expected, IR rates of the ECR strains harboring equivalent mutations in HS1 of *FKS1* and *FKS2* were comparable to that of the susceptible background (WT), and we noted a significant difference between the ECR isolates (Fig. 1B). Intriguingly, ECR isolates with a lower clinical prevalence (those carrying Fks1^R631G^ and Fks2^R665G^) had a lower, but not statistically significant, IR rates at 48 h compared to counterparts with a higher clinical prevalence (carrying Fks1^S629P^ and Fks2^S663P^) (Fig. 1B). Moreover, those ECR isolates did not show a major fitness defect compared to parental WT. Strikingly, the ECR isolates carrying Fks2^F659del^ had a significantly lower IR rate compared to their ECR counterparts carrying Fks2^F659V^ along with the WT (Fig. 1B).

To assess any potential clinical significance, we compared the IR rate of clinical isolates (cECR) carrying Fks2^F659del^ with that of the other ECR isolates. Despite the potential for varied genetic backgrounds represented, clinical ECR isolates carrying Fks2^F659del^ also had lower IR rates compared to the rest of the ECR isolates carrying other mutations (Fig. 1C), implying that Fks2^F659del^ may confer fitness cost irrespective of the background (the sequence type of these ECR clinical isolates carrying Fks2^F659del^ was not determined). Of note, unlike the isogenic isolates, clinical counterparts may also carry additional “suppressor” mutations, which may further alleviate the fitness cost of ECR causing *FKS* mutations.

Next, we assessed the intra-macrophage competition of the iECR isolate carrying Fks2^F659del^ with the WT and Fks2^F659del^ with the ECR counterpart carrying Fks2^F659V^. We engineered green fluorescent protein (GFP)- and red fluorescent protein (RFP)-expressing reporter isolates using protocols described previously (18, 22). Of note, we have previously shown that CBS138 *C. glabrata* cells expressing either GFP or RFP had similar IR rates as compared to the non-fluorescent parental strain (18, 22). THP1 macrophages were infected with cell suspensions containing a mixture of two isolates, and the competitive IR rate was assessed by flow cytometry at 3 h and 24 h pi. Moreover, the supernatant was collected 3 h pi to determine the phagocytosis rate. Consistent with the results of the mono-infection THP1 model, ECR isolates carrying Fks2^F659del^ were outcompeted by the WT (Fig. 1D) and the ECR counterpart carrying Fks2^F659V^ (Fig. 1E). Although the ECR isolate carrying Fks2^F659V^ was outcompeted by the WT, the burden of both isolates stayed constant in the transition from 3 to 24 h, which may indicate that Fks2^F659V^ effectively sustained the competition within the 21-h interval (Fig. 1F). Surprisingly, the isolate harboring Fks2^F659del^ had a higher phagocytosis rate compared to both the WT (Fig. 1G) and its counterpart harboring Fks2^F659V^ (Fig. 1H), whereas Fks2^F659V^ was phagocytosed at a slightly higher rate than the WT (Fig. 1I). Therefore, despite the intracellular Fks2^F659del^ cells were more abundant compared to WT and Fks2^F659V^ during the early hours of intra-macrophage competition, they were still outcompeted by both isolates, which may indicate an intrinsic defect associated with this mutation hindered efficient competition and intracellular replication of Fks2^F659del^ mutants. Collectively, these observations suggest that, except for the significant fitness cost incurred by Fks2^F659del^, iECR isolates showed comparable levels of replication within the macrophage relative to WT.

### *FKS2*, but not *FKS1*, is required for replication inside the macrophages

Fks2 is known to be the main 1,3-β-glucan synthase operational during stress, and β-glucan plays a prominent role in protection against stress (20, 21). Therefore, we hypothesized that a functional *FKS2* is required for replication inside the macrophages. To test this, we generated isogenic *C. glabrata* isolates lacking *FKS1* or *FKS2* and compared their IR rates with the WT strain, using a THP1 macrophage infection assay as described above.

**Fig 2:**
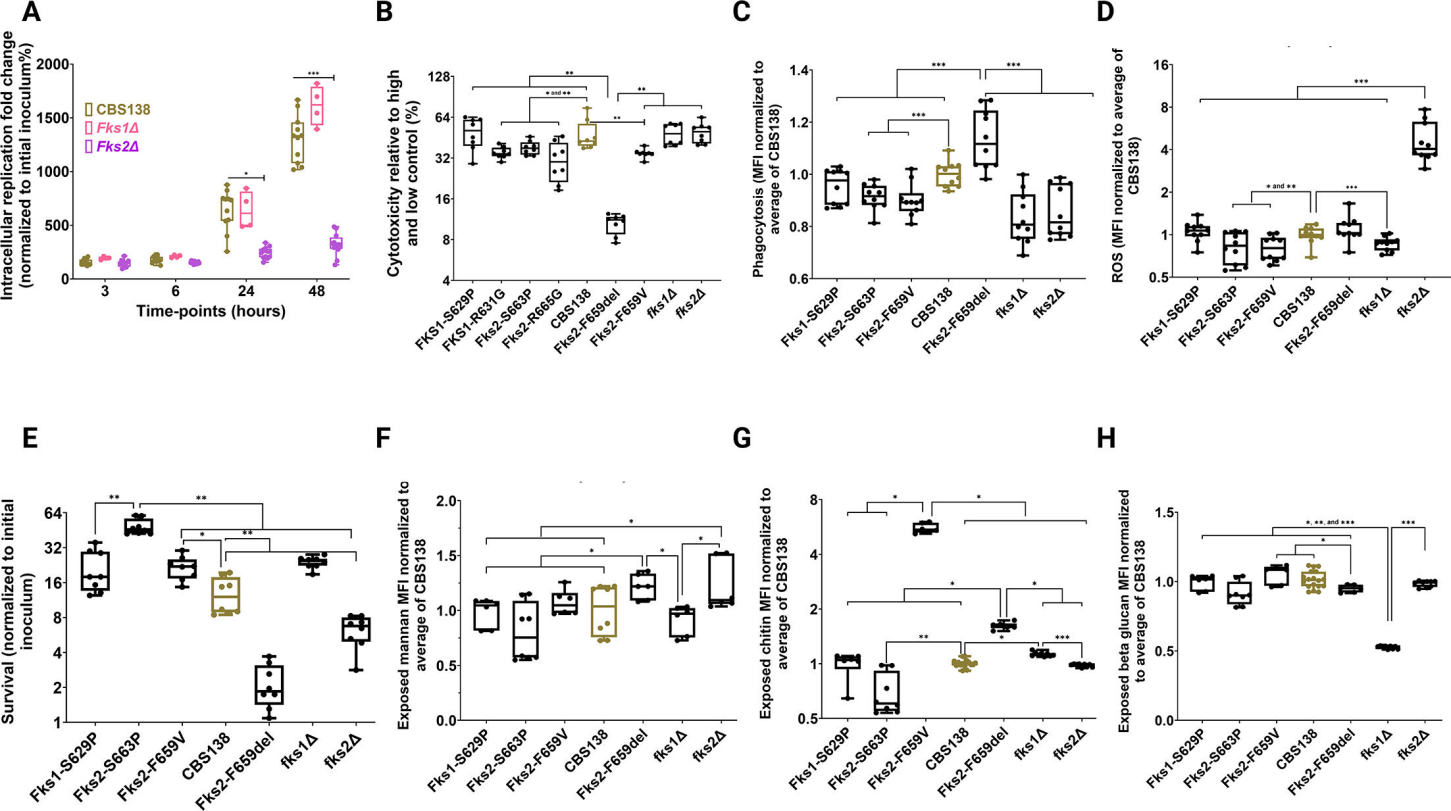
Although *fks1Δ* showed comparable intracellular replication rates relative to wild type within THP1 macrophages, *fks2Δ* mutants showed a severe attenuation of intracellular replication rates (**A**). Fks2^F659del^ induced a significantly lower cytotoxicity to THP1 macrophages compared to wild type (**B**). Fks2^F659del^ and *fks1Δ* mutants had the highest and lowest phagocytosis by primary human neutrophils (**C**), whereas only neutrophils infected with *fks2Δ* mutants produced a significantly higher ROS (**D**). Fks2^F659del^ and *fks2Δ* mutants had the lowest survival during interaction with primary human neutrophils (**E**). Cell wall exposure analysis revealed that Fks2^F659del^ mutants had the highest mannan exposure (**F**), Fks2^F659del^, Fks2^F659V^, and *fks1Δ* exhibited a significantly higher chitin exposure (**G**), and *fks1Δ* showed the least β-glucan exposure (**H**). Each data point reflects an individual biological replicate in all figures. The Wilcoxon test was used for analysis. *, **, ***, and **** refer to *P* values <0.05 and >0.01, <0.01, <0.005, and <0.0005, respectively. THP1: Tohoku Hospital Pediatrics-1.

As predicted, the *fks2Δ* strain showed a severe fitness defect and had a lower IR rate compared to *fks1Δ* and WT, while *fks1Δ* did not show a defect in IR rate compared to WT (Fig. 2A). This observation suggested that Fks2, but not Fks1, is required for replication of *C. glabrata* inside the macrophages.

Given the significant defect in the IR rate of the *fks2Δ* and the ECR isolate carrying Fks2^F659del^, we wondered whether these isolates incur less damage to infected macrophages compared to other isolates. For this, THP1 macrophages were infected at an MOI of 5/1, and damage incurred to macrophages was measured via a lactate dehydrogenase kit (Sigma) at 24 h pi. Except for the ECR-Fks1^S629P^, the rest incurred significantly lower damage to macrophages, and the ECR-Fks2^F659del^ incurred the least damage to macrophages, which is consistent with its lower IR rate within macrophages (Fig. 2B). Surprisingly, macrophages infected with *fks2Δ* had comparable damage relative to those infected with *fks1Δ* and susceptible isolates (Fig. 2B). This discrepancy in inducing macrophage damage incurred by Fks2^F659del^ and *fks2Δ* was surprising since *C. glabrata*-induced macrophage damage had been linked to the IR rate (23). Nonetheless, differential changes in the architecture of the cell wall may trigger inflammasome activities by *fks2Δ*, which consequently exerted a higher macrophage damage (24–26). Altogether, these observations suggested that (i) Fks2 is required to establish infection within macrophages, (ii) most ECR mutants incurred a lower damage to macrophages, and (iii) despite the similarity of the IR defect of Fks2^F659del^ and *fks2Δ*, only macrophages infected with Fks2^F659del^ incurred a significantly lower damage than those infected with the remaining mutants.

### Fks2^F659del^ and *fks2Δ* mutants are more effectively killed by primary human neutrophils

Neutrophils are among the first innate immune cell responders during fungal infections, and neutropenic patients are highly prone to acquire candidemia due to *C. glabrata* (27). Therefore, we sought to determine the fitness of the iECR mutants (Fks1^S629P^, Fks2^S663P^, Fks2^F659V^, and Fks2^F659del^), *fks1Δ* and *fks2Δ*, and WT during interaction with primary human neutrophils collected from two healthy donors.

Although neutrophils exert multiple fungicidal effectors, phagocytosis and the production of reactive oxygen species (ROS) are known as the most potent fungicidal effectors (28). Therefore, we assessed phagocytosis rate and reactive oxygen (ROS) production by neutrophils using a multiparametric flow cytometry-based approach 1 h pi (MOI = 3/1) and measured fungal survival by plating and CFU counting 4 h pi (MOI = 1/3) (see Materials and Methods). To measure ROS, we treated CellMask Deep Red-stained neutrophils with dihydrorhodamine (DHR) 123, and the MFI of DHR was gated based on uninfected controls. Survival was calculated by normalization of CFU after 4 h against the CFU prior to infection.

Similar to the results observed in macrophages, Fks2^F659del^ (but not *fks2Δ*) and *fks1Δ* had the highest and lowest phagocytosis rates by neutrophils, respectively (Fig. 2C). ECR mutants carrying Fks2^S663P^ and Fks2^F659V^ and *fks1Δ* and *fks2Δ* were less phagocytosed compared to WT. Consistent with their higher phagocytosis, neutrophils infected with ECR mutants carrying Fks2^S663P^ and Fks2^F659V^ and *fks1Δ* produced a lower ROS compared to WT (Fig. 2D). Nonetheless, neutrophils infected with *fks2Δ* produced a significantly higher ROS than all strains tested despite being less phagocytosed to a lower extent than WT. We also noted that despite the higher phagocytosis of ECR-Fks2^F659del^, neutrophils infected with this strain produced a similar ROS level compared to WT. Interestingly, Fks2^S663P^ and Fks2^F659del^ had the highest and the lowest survival during interaction with neutrophils compared to all strains tested (Fig. 2E), which is consistent with their phagocytosis level. The rest of the ECR mutants had either similar (Fks2^S629P^) or higher survival (Fks2^F659V^, which again compares favorably with their phagocytosis level [Fig. 2E]). Consistent with observations made with macrophages, *fks1Δ* did not show a fitness defect (here even had a higher survival) compared to WT during interaction with neutrophils, whereas *fks2Δ* had a significantly lower survival (Fig. 2E). Collectively, these observations suggest that Fks2^F659del^ and *fks2Δ* are more susceptible to killing by primary human neutrophils, whereas other ECR mutants and *fks1Δ* either show comparable or higher survival during co-incubation with neutrophils.

### Differential phagocytosis of mutants by innate immune cells could be due to alteration of cell wall exposure

*FKS* mutations are known to alter the catalytic capacity of β-glucan synthase (12), which consequently may trigger the cell wall integrity pathway to produce higher levels of chitin and mannan to compensate for β-glucan loss. Such cell wall remodeling is known to impact recognition by phagocytes (29, 30) through antigen unmasking via interaction with multiple pathogen-associated molecular patterns (PAMPs) on the fungal cell surface (29, 30). Therefore, we sought to assess the exposure of mannan, β-glucan, and chitin in the aforementioned mutants and WT using concanavalin A AF647, wheat-germ agglutinin-FITC, and ant-β-glucan antibodies-AF647, respectively (31, 32).

Since Fks2^F659del^ and *fks2Δ* behaved similarly during interaction with both innate immune cells, we speculated that cell wall remodeling was similar between these mutants. Indeed, both strains had the highest level of mannan exposure compared to the rest of the isolates tested (Fig. 2F). Although the higher mannan exposure may explain the higher phagocytosis of Fks2^F659del^ as suggested previously (31, 33), it does not explain the similar phagocytosis of *fks2Δ*. This likely reflects the fact that mannan is an extremely complex cell wall component (containing O-linked, phospholipo-mannan, phosphor-mannan, α−1,6, α−1,2, and β−1,2 mannan, etc.) (29). The overall mannan and cell wall composition might differ between the two strains, with Fks2^F659del^ and Fks2^F659V^ mutants showing the highest chitin exposure, whereas Fks2^S663P^ displayed the lowest chitin exposure compared to all strains tested (Fig. 2G). It is known that ECR mutants may have higher compensatory levels of chitin (14). While chitin can confer an immunomodulatory impact (14, 29, 30), this did not appear to be the case for Fks2^F659del^, since it was highly susceptible to neutrophils and macrophages. β-glucan exposure is a prominent determinant for phagocytosis of fungal pathogens (29, 30). Consistent with a lower phagocytosis of *fks1Δ* by neutrophils, this mutant had the lowest β-glucan exposure compared to all strains tested, but Fks2^F659del^ had a higher phagocytosis by neutrophils and macrophages and had a lower β-glucan exposure at least compared to WT (Fig. 2H). Collectively, our flow cytometry-based assay supports Fks2^F659del^ and Fks2^F659V^ having similar cell wall antigen exposure reflective of cell wall remodeling, which was clearly distinct from that of *fks2Δ*. Future studies are needed to provide a higher resolution cell wall architecture among these mutants and the WT.

### Sensitivity to multiple stresses underlies the lower survival of Fks2^F659del^ during interaction with macrophages and neutrophils

**Fig 3:**
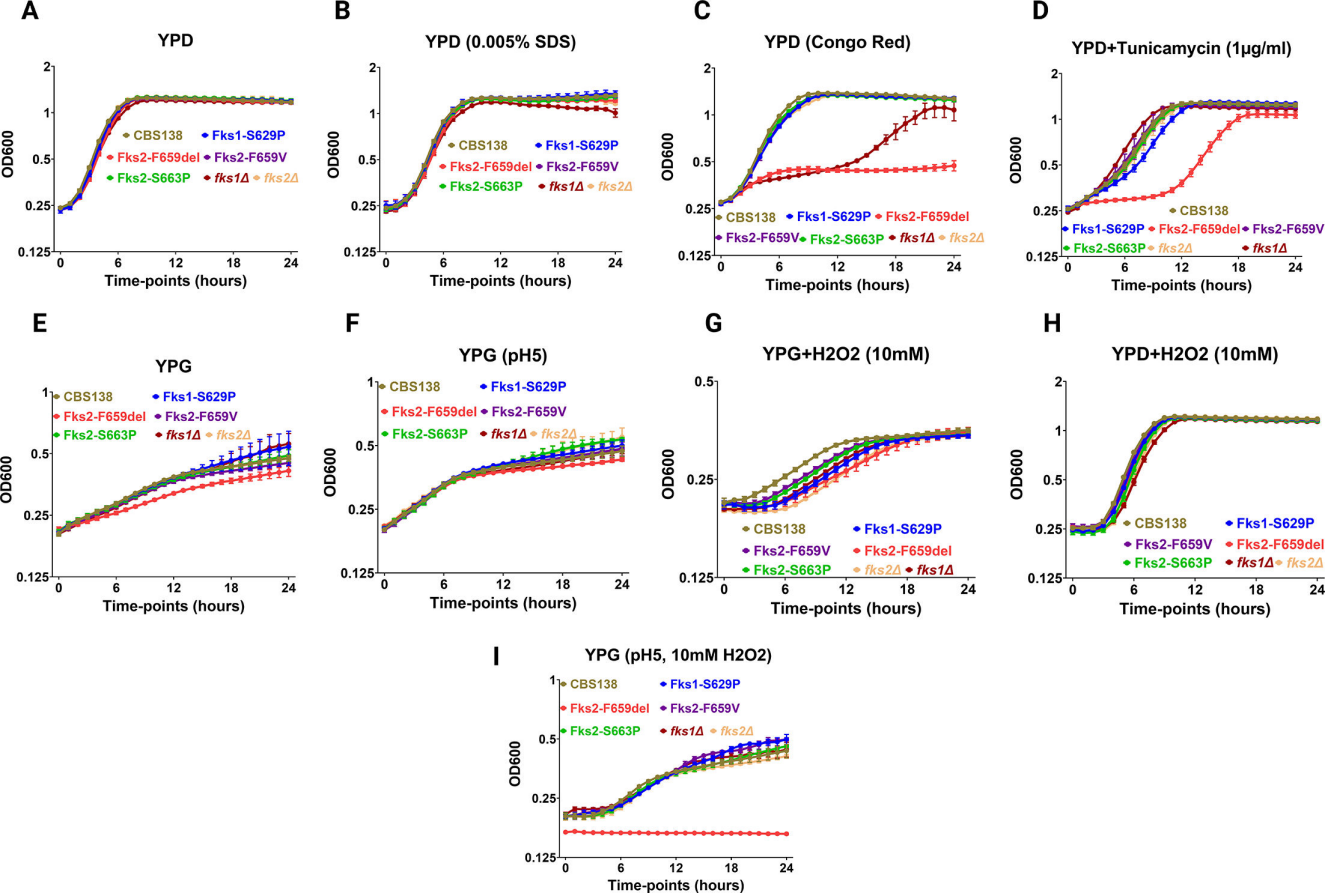
Kinetic *in vitro* growth assessment of isogenic ECR and wild type during incubation in YPD (**A**), SDS (**B**), Congo Red (**C**), endoplasmic reticulum stress (**D**), YPG (**E**), pH 5 YPG (**F**), YPG + 10 mM H_2_O_2_ (**G**), YPD + 10 mM H_2_O_2_ (**H**), pH 5 YPG + 10 mM H_2_O_2_ (**I**). At least three independent biological replicates were used for each strain under each condition tested.

Aside from modulating interactions with innate immune cells, cell wall remodeling may also impact susceptibility to various host-derived stresses. To identify the prominent stresses underlying intracellular susceptibility of Fks2^F659del^ and *fks2Δ*, we set out to assess the *in vitro* growth rate of above mutants and WT in the absence (yeast-peptone-dextrose or YPD) and presence of various stresses, including cell wall stress (Congo Red and sodium dodecyl sulfate [SDS]), alternative carbon source (yeast-peptone-glycerol or YPG), oxidative stress (YPD + 10 mM H_2_O_2_ and YPG + 10 mM H_2_O_2_), low pH (pH 5 YPG), endoplasmic reticulum stress (YPD + 1 µg/mL Tunicamycin), and phagocyte-like condition (pH 5 YPG + 10 mM H_2_O_2_). Although the growth rate of mutants was comparable to that of WT in YPD (Fig. 3A), the differences were more prominent in the presence of stressors. *fks1Δ* was the only mutant showing a slight growth defect to SDS (Fig. 3B). Nonetheless, Fks2^F659del^, followed by *fks1Δ,* showed the highest growth defects, respectively, during Congo Red (Fig. 3C). The fks*2Δ* mutant did not show any obvious growth defects related to cell wall stress when compared to *fks1Δ* and Fks2^F659del^ (Fig. 3B and C). Although higher growth defects of *fks1Δ* during cell wall stresses may be speculated to result from a thinner β-glucan layer when compared to *fks2Δ* (as shown in our cell wall analysis), this assumption may not justify the extreme growth attenuation of Fks2^F659del^ in the presence of a functional Fks1 allele.

Similarly, the Fks2^F659del^ mutant showed the highest growth defect with ER stress, whereas the Fks1^S629P^ mutant only showed a slight growth attenuation at early hours (Fig. 3D). Fks2^F659del^ also showed the highest growth defect in YPG (Fig. 3E), whereas, curiously, this growth defect was offset in pH 5 YPG, and all mutants showed growth levels comparable to WT (Fig. 3F). Although mutants and WT showed similar final growth rates during incubation in YPG supplemented with H_2_O_2_ (Fig. 3G), the growth rate of mutants was significantly impaired relative to WT during the initial 14 h, with Fks2^F659del^ and *fks2*Δ showing the most prominent growth defect in those early hours. Nonetheless, except for *fks1Δ*, all mutants showed growth rates comparable to WT during incubation in YPD supplemented with H_2_O_2_ (Fig. 3H). Therefore, oxidative stress exerted a more deleterious effect in YPG. Except for mutant Fks2-F659del, which was not able to replicate in pH 5 YPG supplemented with H_2_O_2_, the remaining mutants had comparable growth rates to WT (Fig. 3I). Again, *fks2Δ* did not mimic the growth defects manifested by Fks2^F659del^, which further substantiates the hypothesis that the nature of cell wall remodeling and the mechanisms underlying this susceptibility are potentially very distinct between these two mutants. Collectively, our *in vitro* growth rate analysis suggests that susceptibility to multiple host-derived stresses may corroborate the lower intracellular fitness of Fks2^F659del^ during interaction with macrophages and neutrophils.

### The higher susceptibility of Fks2^F659del^ is due to higher basal ROS levels and the inability to mount an adaptive oxidative stress response

**Fig 4:**
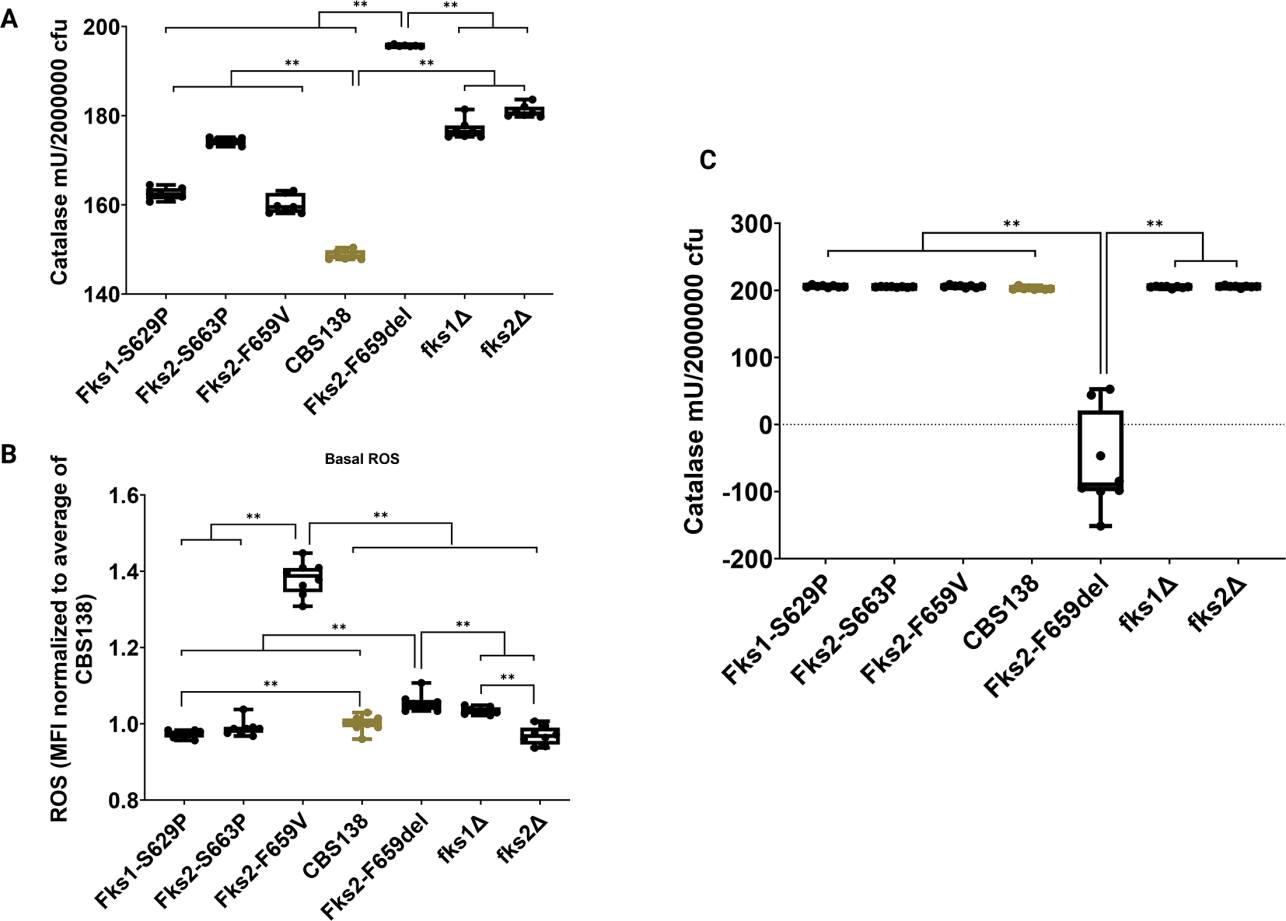
Basal catalase level of mutants and wild type after exposure to 40 µM of H_2_O_2_ for 30 min measured by Amplex Red (**A**). The basal ROS level of mutants and wild type was measured using dihydrorhodamine 123 (**B**). Adaptive catalase level of mutants and wild type after exposure to 10 mM of H_2_O_2_ for 360 min, measured by Amplex Red (**C**). The Wilcoxon test was used for analysis. *, **, ***, and **** refer to *P* values <0.05 and >0.01, <0.01, <0.005, and <0.0005, respectively. ROS: reactive oxygen species.

Since Fks2^F659del^ and *fks2Δ* mutants had the most attenuated growth rates during incubation in YPG supplemented with 10 mM H_2_O_2_ and were highly susceptible to both macrophages and neutrophils, we speculated that these mutants are unable to mount a robust oxidative stress response (OSR) during phagocytosis. Therefore, as a proxy of oxidative stress response, we measured the catalase level of mutants and WT during exposure to H_2_O_2_ using Amplex Red (see Materials and Methods) at basal (exposure to 40 µM of H_2_O_2_ for 30 min) and adaptive (exposure to 10 mM of H_2_O_2_ for 360 min) levels. The 10 mM H_2_O_2_ challenge was chosen as it exerted the highest defect in both Fks2^F659del^ and *fks2Δ* in YPG. All mutants had a significantly higher catalase level than the WT, which was highest in mutants Fks2^F659del^ and *fks2Δ* (Fig. 4A). The higher catalase activity of mutants prompted us to hypothesize that they may have a higher basal ROS level likely induced by the underlying mutation. Therefore, we measured the basal ROS using dihydrorhodamine 123 (DHR123), and the MFI values were normalized against those of WT. There was not a clear relationship between basal ROS and catalase levels for some mutants, since *fks2Δ* and Fks1^S629P^, despite having high basal catalase levels, had a significantly lower basal ROS levels (Fig. 4B). Nonetheless, both Fks2^F659V^ and Fks2^F659del^ had the highest basal ROS and also had a high basal catalase level (Fig. 4B). Next, we assessed the adaptive response. Interestingly, whereas Fks2^F659del^ showed an extremely poor adaptive OSR, the rest of the mutants detoxified H_2_O_2_ as effectively as WT (Fig. 4C). These results further support the notion that fitness defects observed with Fks2^F659del^ and *fks2Δ* may reflect distinct physiological dysregulations. Collectively, these experiments suggest that Fks2^F659del^ susceptibility to innate immune cells is enhanced by an inability to mount an effective adaptive OSR. Notably, our whole-genome sequencing analysis indicated that the severe fitness defect of the Fks2^F659del^ may not be explained by additional genomic changes deleteriously impacting the open reading frame of genes involved in key cellular functions.

### *C. glabrata*-macrophage transcriptomic responses

To gain a mechanistic and holistic view during interaction with THP1 macrophages, we performed dual RNA-seq from THP1 macrophages infected with CBS138 and isogenic mutants derived from it, that is, *fks1Δ, fks2Δ*, Fks2^F659del^, and Fks2^F659V^. We chose these isolates as none of the other ECR mutants, such as Fks2^S663P^, etc., showed any fitness cost compared to WT, whereas we observed a profound phenotypic difference between the other pairs (*fks1Δ* vs *fks2Δ* and Fks2^F659del^ vs Fks2^F659V^) and with WT. Moreover, gaining a mechanistic understanding of the intracellular fitness defect of Fks2^F659del^ may unravel novel druggable targets aiming to curb the intracellular fitness of *C. glabrata*. Macrophages were infected with MOI 5/1, extensively washed at 3 h pi, and fresh RPMI was added, followed by RNA extraction at 3 and 24 h pi.

We first explored the overall transcriptional landscape of all *C. glabrata* isolates by using principal component analysis (Fig. S1A). Despite a considerable variance of samples within time-points and strains, we still observed both time- and strain-dependent differences between the studied *C. glabrata* samples.

**Fig 5:**
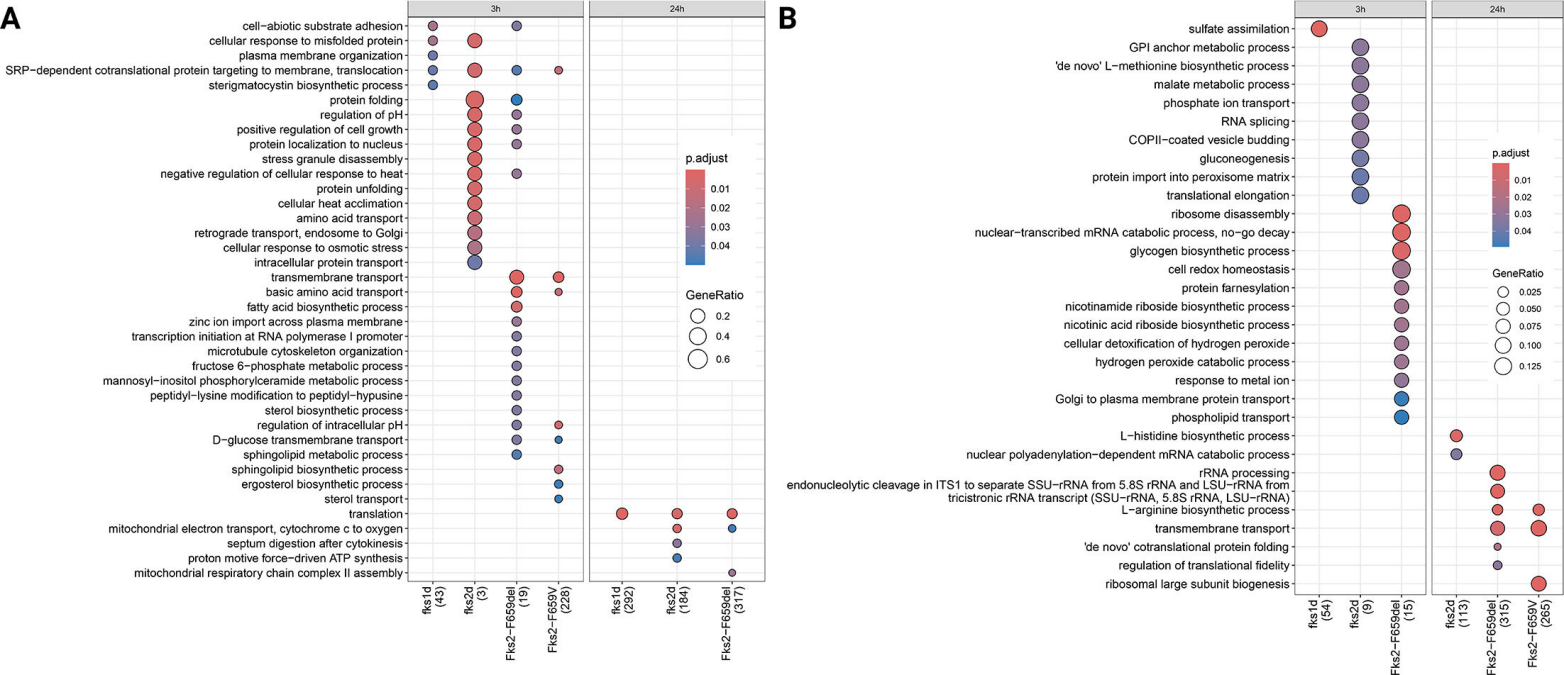
Transcriptomic analysis of *C. glabrata* strains. GO term enrichment analysis (category “Biological Process”) of differentially expressed genes of *C. glabrata* strains compared to the strain CBS138, which demonstrates upregulated (**A**) and downregulated (**B**) pathways. The numbers near each comparison correspond to the “counts” of clusterProfiler (i.e., total number of genes assigned to GO categories). GeneRatio corresponds to the ratio between the number of input genes assigned to a given GO category and “counts.” Only significant (*P*adj < 0.05) enrichments are shown. Adjustment of *P*-values is done by the Benjamini–Hochberg procedure. Comparisons where no significant enrichments were detected are not displayed.

By using differential gene expressions and gene ontology (GO) term enrichment analyses, we further dissected and explored the transcriptional patterns of different *C. glabrata* mutants compared to WT (Fig. 5B and C; Table S2). We first focused on upregulated pathways (Fig. 5A). Protein folding and pH regulation were among the common upregulated pathways enriched in both Fks2^F659del^ and *fks2Δ* mutants. Upregulated genes enriched for biosynthetic processes, such as fatty acids and sterols, were exclusive to the Fks2^F659del^ mutant and particularly interesting since such processes are heavily energy-consuming (34, 35), and they were shown to be downregulated by *C. glabrata* during macrophage infection (36). Upregulated pathways between the two strains showed more similarities at 24 h pi and converged on translation and mitochondrial processes (Fig. 5A; Table S2). We next focused on GO term analysis to identify downregulated pathways specifically enriched for mutants compared to WT (Fig. 5B). Interestingly and consistent with failure to induce H_2_O_2_ detoxification, Fks2^F659del^ mutants were enriched for pathways involved in cell-redox homeostasis, cellular detoxification of hydrogen peroxide, hydrogen peroxide catabolic processes, among the others (Fig. 5B). ROS detoxification is vital for intracellular adaptation and survival (37, 38), and this dysregulated ROS stress response during macrophage infection may underscore the lower intracellular fitness of Fks2^F659del^ mutant. Go term analysis of *fks2Δ* mutant, on the other hand, revealed enrichment for downregulated pathways involved in GPI-anchor and malate metabolic processes as well as gluconeogenesis and protein import into peroxisome matrix. Interestingly, the latter processes are vital for intracellular pathogenesis of *Histoplasma capsulatum* (39, 40). Collectively, our RNAseq observations suggest that the intracellular susceptibility of Fks2^F659del^ and *fks2Δ* mutants is divergent and is potentially due to dysregulated OSR and metabolic responses, respectively. Contrary to intracellular *C. glabrata* cells, macrophages tended to mount more similar responses, with a few exceptions (Fig. S1B through D, S2A and B). Macrophage RNAseq data can be downloaded using the following link: https://github.com/Gabaldonlab/C_glabrata_MDR.

### ECR isolates carrying Fks2^F659del^ are outcompeted by counterparts harboring Fks2^F659V^ and the susceptible parent in the context of gut colonization

Given that *C. glabrata* is a GI tract commensal and that the ECR isolates are thought to originate from the gut (41, 42), we performed GI-tract competition involving CBS138, *fks1Δ, fks2Δ*, and ECR isolates carrying Fks2^F659del^ and Fks2^F659V^ for reasons indicated above.

Gut colonization was induced by oral gavage using suspensions containing a mixture of two isolates, one expressing GFP and one non-fluorescent. We previously demonstrated that GFP expression incurs a slight fitness cost compared to non-fluorescent counterparts. Fecal samples collected on days 1, 3, 5, and 7 were plated on YPD agar plates, and after 48-h incubation at 37°C, the GFP and non-fluorescent *C. glabrata* colonies were visualized by Typhoon.

**Fig 6:**
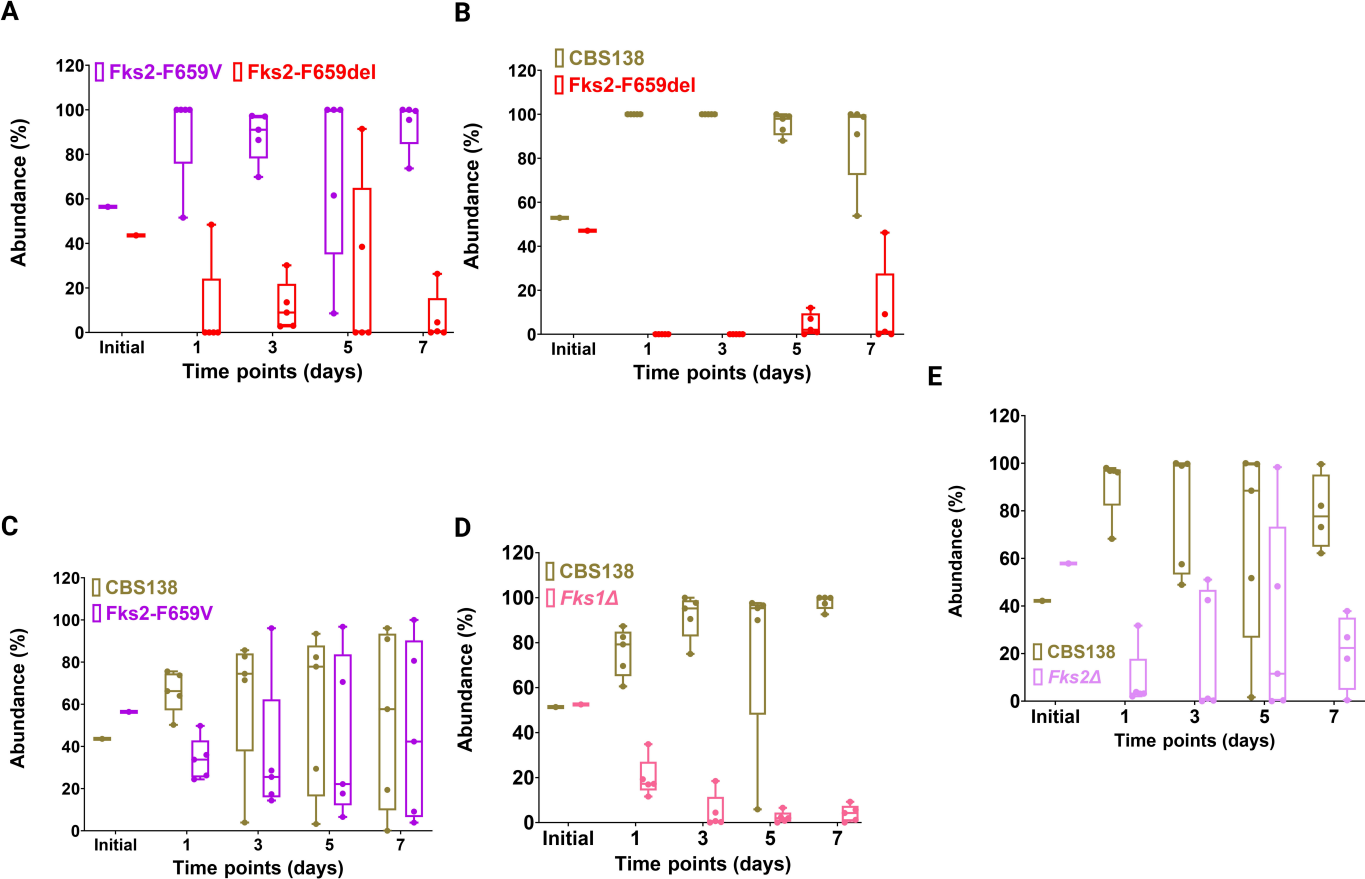
Competitive fitness of Fks2^F659del^ vs Fks2^F659V^(**A**) and Fks2^F659del^ vs wild type (**B**), Fks2^F659V^ vs wild type (**C**), *fks1Δ* vs wild type (**D**), and *fks2Δ* vs wild type (**E**) during gastrointestinal tract colonization. Each data point reflects an individual biological replicate (one mouse) in all figures.

Expectedly, the ECR isolate harboring F659del was readily outcompeted by the ECR counterpart carrying Fks2^F659V^ and the susceptible WT isolate (Fig. 6A and B). The competition between the susceptible isolate and the ECR harboring Fks2^F659V^ revealed a similar competence at the last time-point (day 7) (Fig. 6C). Next, we assessed the competence of *fks1Δ* and *fks2Δ* for gut colonization when compared to the susceptible parent. Intriguingly, both deletants were readily outcompeted by CBS138 (Fig. 6D and E), which suggests that both *FKS1* and *FKS2* are required for robust fitness and efficient gut colonization. Altogether, these observations revealed that ECR isolates carrying Fks2^F659del^ are unfit in the context of gut colonization, whereas the ECR harboring Fks2^F659V^ efficiently colonizes the GI tract. Moreover, efficient gut colonization by *C. glabrata* requires functional *FKS1* and *FKS2* genes.

### ECR isolates carrying Fks2^F659del^ are outcompeted by counterparts harboring Fks2^F659V^ and the susceptible parent in the context of systemic infection

To further explore the clinical relevance of our findings, we performed an *in vivo* murine systemic infection model involving the same set of isolates used in our gut colonization model. Mice were infected systemically with a mixture of two isolates using the rhino-orbital route, and each competition arm included 12 mice and three time-points, days 1, 4, and 7. Kidney and liver collected at each designated time-point were extensively homogenized and plated on YPD agar plates for CFU counting. Colonies were visualized using Typhoon.

**Fig 7:**
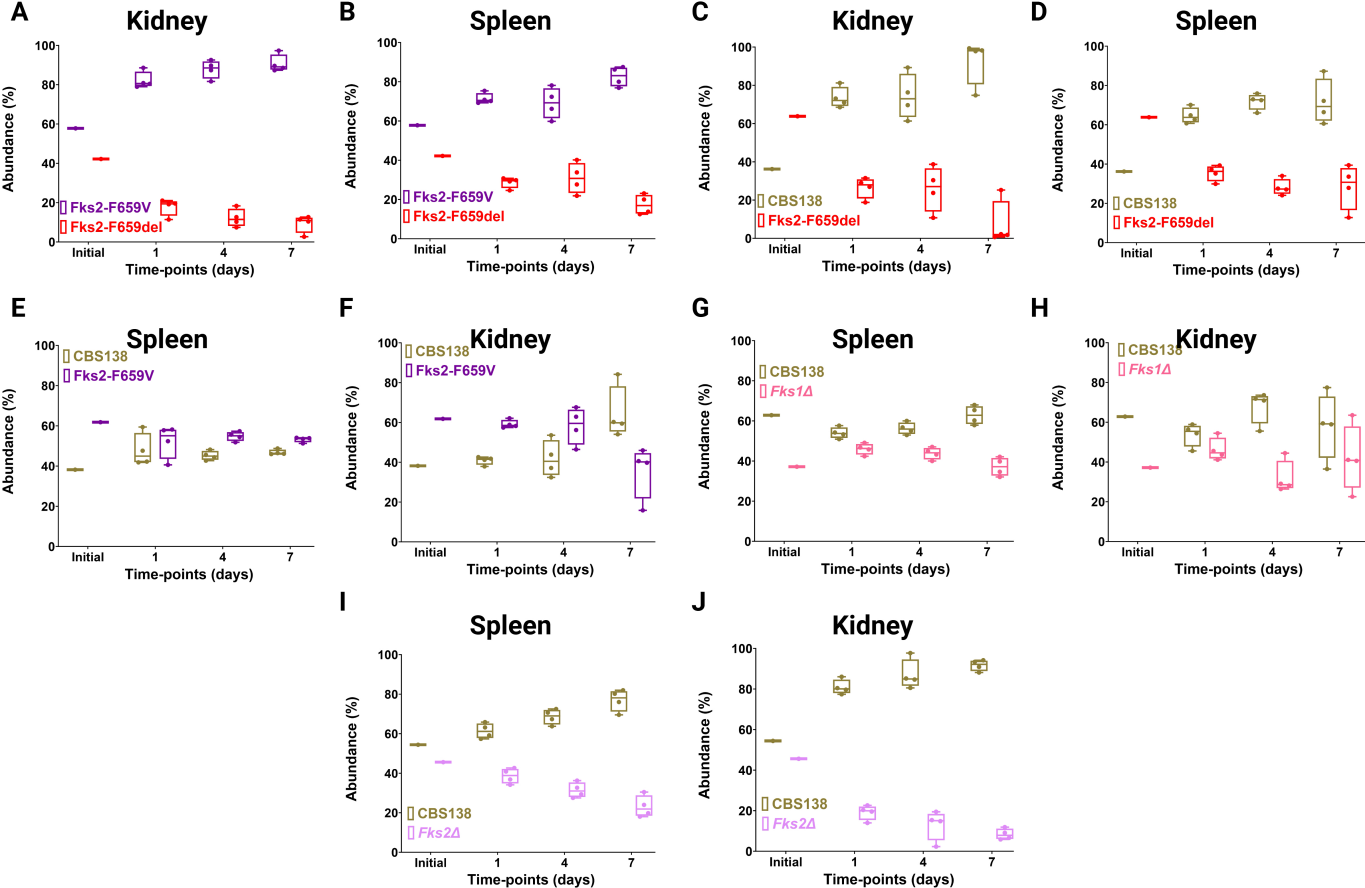
Competitive fitness of Fks2^F659del^ vs Fks2^F659V^ in kidney (**A**), Fks2^F659del^ vs Fks2^F659V^ in spleen (**B**), Fks2^F659del^ vs wild type in kidney (**C**), Fks2^F659del^ vs wild type in spleen (**D**), and Fks2^F659V^ vs wild type in spleen (**E**), and Fks2^F659V^ vs wild type in kidney (**F**), *fks1Δ* vs wild type in spleen (**G**), *fks1Δ* vs wild type in kidney (**H**), *fks2Δ* vs wild type in spleen (**I**), and *fks2Δ* vs wild type in kidney (**J**) in the context of systemic infection. Each data point reflects an individual biological replicate (one mouse) in all figures.

Similar to our *ex vivo* macrophage and gut colonization mice model, ECR isolate carrying Fks2^F659del^ was outcompeted by the ECR counterpart carrying Fks2^F659V^ and the susceptible parental isolate in both kidney and spleen (Fig. 7A through D). Interestingly, WT and the ECR carrying Fks2^F659V^ competed comparably in spleen (Fig. 7E), whereas the latter was outcompeted by the susceptible isolate in kidney (Fig. 7F). The *fks1Δ* mutant comparably competed with the susceptible isolate in both spleen and kidney (Fig. 7G and H), which may suggest that *FKS1* is not required for establishing systemic infection. *Fks2Δ* mutant, however, was readily outcompeted by the susceptible isolate in both spleen and kidney (Fig. 7I and J), which might be an indication of the requirement of *FKS2* to effectively establish systemic infection. Collectively, our *in vivo* systemic infection competition indicated that the ECR isolates carrying Fks2^F659del^ are outcompeted by other ECR and susceptible isolates, whereas the persistence of the ECR isolate carrying Fks2^F659V^ varies depending on the infected organ. Moreover, to effectively establish systemic infection, *C. glabrata* requires active Fks2, but not Fks1.

## DISCUSSION

The hypothesis that severe fitness costs thwart the clinical prevalence of ECR in *C. albicans* begs the question of why ECR *C. glabrata* isolates are more prevalent in the clinic? Additionally, whereas both *FKS1* and *FKS2* are operational in *C. glabrata*, their importance during commensalism and infection has not been determined. Herein, we show that most ECR mutants, except Fks2^F659del^, have comparable fitness relative to susceptible WT during macrophage and neutrophil infection. Moreover, Fks2^F659del^ mutants are less fit during GI-tract commensalism and systemic infection compared to Fks2^F659V^ and susceptible WT. Interestingly, while both *FKS1* and *FKS2* are important for robust induction of gut commensalism, only *FKS2* was important during interaction with innate immune cells and systemic infection. The lower fitness of Fks2^F659del^ and *fks2Δ* mutants during interaction with macrophages was potentially associated with dysregulated transcriptomic OSR and metabolic responses, respectively. Collectively, our study shows that being refractory to echinocandins through the acquisition of key *FKS* mutations did not impede the fitness of most ECR mutants during interaction with frontline innate immune cells, which potentially underscores the notable prevalence of ECR *C. glabrata* in the clinic. Therefore, such ECR mutants may thrive in diverse host niches even in the absence of echinocandins. Finally, differential amino acid substitutions at the same position may exert a distinct impact on fitness in diverse ecological niches within the host and have the potential to unravel novel druggable target/s aiming at curbing the intracellular fitness of *C. glabrata*.

We previously showed that the intracellular fitness defect of FLZR *C. glabrata* isolates can be rescued by acquisition of *FKS* mutations. The extent of such rescue depended on the mutation type and *FKS* allele, with prevalent *FKS* mutations, such as Fks1^S629P^ and Fks2^S663P^ vs Fks1^R631G^ and Fks2^R665G^, and *FKS2* mutants generally better rescued the intracellular replication within THP1 macrophages (18). Herein, we extended our previous study and not only investigated such mutants in a susceptible background but also examined how mutations at a single residue, that is, Fks2^F659V^ vs. Fks2^F659del^, impact fitness. Expectedly, ECR mutants carrying Fks1^S629P^, Fks2^S663P^, Fks1^R631G^, and Fks2^R665G^ did not show significant fitness cost within THP1 macrophages when compared to susceptible WT regardless of the background (clinical or laboratory-made isogenic strains). Intriguingly, whereas Fks2^F659V^ showed a similar intracellular replication rate relative to susceptible WT and other isogenic ECR isolates, Fks2^F659del^ isolates were more phagocytosed and showed a dramatic intracellular replication defect irrespective of the background (clinical or isogenic). Consistently, *fks2Δ* mutants showed similar intracellular replication defects, whereas *FKS1* seemed dispensable for replication within THP1. Although *C. glabrata*-induced macrophage lysis is speculated to be linked to intracellular replication rate, only Fks2^F659del^ mutants (23, 34), but not *fks2Δ*, significantly attenuated the virulence as judged by LDH level. This may indicate that other intracellular replication-independent factors from *C. glabrata* side, such as secretory proteins, or induction of certain pathways from the host side, such as inflammasome, may underlie comparable virulence of *fks2Δ* relative to WT. Consistent with observations made with THP1 macrophages, Fks2^F659del^ and *fks2Δ* mutants had significantly lower survival rates during interaction with primary human neutrophils, whereas *fks1Δ* and Fks2-S663P had significantly higher survival relative to susceptible WT background. Of note, the higher survival of *fks1Δ* relative to susceptible WT is not necessarily an indication of higher fitness during neutrophil interaction, as this mutant was less efficiently phagocytosed. Nonetheless, the dispensability of *FKS1* during interaction with both macrophages and neutrophils is consistent with the observations that Fks1 is the main β-glucan synthase during homeostasis, whereas Fks2 is the main β-glucan synthase during stress encounters (21). These observations collectively suggest that most ECR mutants, except Fks2^F659del^, had comparable fitness relative to susceptible WT during interaction with innate immune cells and that *FKS2* plays a prominent role in intracellular survival.

The severe fitness defect of Fks2^F659del^ and *fks2Δ* during interaction with innate immune cells might converge on an inability of such mutants to mount certain responses to a similar set of stresses. Indeed, Fks2^F659del^ mutants were susceptible to cell wall and ER stresses and, interestingly, could not replicate in macrophage-like media. Whereas *fks2Δ* only showed growth defect in YPG supplemented with H_2_O_2_, these *in vitro* growth rate observations may indicate that, despite similar fitness defects during interaction with innate immune cells, Fks2-F659del and *fks2Δ* could predispose susceptibility to various stresses, which may trigger divergent stress responses. Consistent with our *in vitro* growth rate observations, our PCA plot and pathway enrichment analysis showed that the Fks2^F659del^ mutant mounted a distinct transcriptomic response than did the *fks2Δ,* especially at 3 h pi within THP1 macrophages. Intriguingly, the downregulated pathways specifically enriched for *fks2Δ* involved metabolic processes, such as gluconeogenesis and peroxisome assembly, which are known as pivotal determinants underlying robust induction of intracellular lifestyle in another intracellular fungal pathogen, *Histoplasma capsulatum* (39, 40). Therefore, metabolic retardation might be among the major factors conferring attenuated fitness of *fks2Δ* during interaction with macrophages. Nonetheless, ROS detoxification and redox homeostasis were among the major enriched pathways downregulated by Fks2-F659del at 3 h pi. This was a surprising observation since ROS is a major stress imposed by macrophages upon pathogen engulfment, and the hypersusceptibility of Fks2^F659del^ mutants to H_2_O_2_ should hypothetically result in enrichment of upregulated pathways conferring resistance to ROS. Interestingly, observations made in *Saccharomyces cerevisiae* indicate that the lower concentrations of H_2_O_2_ (0.2 mM) induce the OSR pathway and genes involved in canonical redox balance, whereas the higher and overwhelming concentrations of H_2_O_2_ (2 mM) induce a very different set of transcripts (43). Keeping with this observation, the overwhelming intracellular ROS experienced by Fks2^F659del^ may have contributed to this dysregulated OSR response. Understanding the link between failure in mounting OSR and Fks2^F659del^ warrants future investigation since our whole-genome sequencing data did not find candidate mutations impairing key cellular functions, such as transcription factors regulating OSR. This overwhelming ROS may damage other key macromolecules, including lipids, proteins, and DNA/RNA, and further impair homeostasis during ROS. Consistently, the RNAseq of intracellular Fks2^F659del^ was enriched for upregulated pathways involved in fatty acid and sterol biosynthesis 3 h pi. Since such anabolic processes require extensive resource allocation and are costly (35), WT *C. glabrata* is known to downregulate such processes during the initial stages of the intracellular lifestyle (36) and, similar to numerous pathogens, has evolved strategies to scavenge and rely on fatty acid and sterol produced by the host (34, 35). Nonetheless, uptake of such macromolecules from the host might not be sufficient to repair the extensive membrane damage in the presence of high oxidative stress and dysregulated OSR at 3 h pi, and Fks2^F659del^ requires biogenesis of such macromolecules to fulfill survival within macrophages. Consistently, macrophages infected with Fks2^F659del^ are enriched for downregulated pathways involved in lipid and cholesterol storage. Furthermore, macrophages infected with Fks2^F659del^ and *fks2Δ* lack enrichment for the upregulated pathway of “response to hypoxia” at 3 h pi. Therefore, these observations from the host side may indicate that macrophages sense the ability to contain such mutants and therefore mount a “more relaxed” response. Collectively, our RNAseq study identified dysregulated metabolic and oxidative stress responses underlying intramacrophage fitness defects of *fks2Δ* and Fks2^F659del^, respectively.

Since *C. glabrata* is a major cause of candidemia (2) and the GI tract is believed to be a primary reservoir of ECR in patients (11, 41, 42), we were interested in examining the fitness of ECR mutants and the importance of *FKS1* and *FKS2* in the context of GI-tract colonization and systemic infections. Expectedly, Fks2^F659del^ was outcompeted by both Fks2^F659V^ and CBS138 in the GI tract and systemic infection in all organs tested, that is, spleen and kidney, which we have shown to be a permissive environment for retention of ECR mutants (18). The combination of our *in vivo* and *ex vivo* analysis, therefore, potentially highlights why patients infected with Fks2^F659del^ may have a better prognosis (10). Of note, it is also possible that some ECR isolates carrying Fks2^F659del^ may gain additional mutations during the course of infection to offset the severe fitness defect arising from this mutation.

Our *in vivo* data also indicated that both *FKS1* and *FKS2* are important for stable induction of GI-tract colonization. Nonetheless, only *FKS2* was important for robust induction of systemic infection, which is consistent with our *ex vivo* observations and that *FKS2* is the major β-glucan synthase during stress encounter, while *FKS1* is more prominent during homeostasis. This observation is intriguing since, unlike systemic infection favoring *FKS2* activation, the complexity of the GI tract environment enforces *C. glabrata* to activate both *FKS1* and *FKS2* for effective colonization. Although it may not necessarily represent *C. glabrata*, studies on *C. albicans* showed that the β-glucan exposure level is inversely related to gut colonization, that is, the higher the β-glucan exposure, the lower the ability to colonize the GI tract (44). Nonetheless, despite a lower β-glucan exposure, *fks1Δ* mutants could not sustain the GI-tract colonization. This lack of sustainability in our study could not be due to elements of microbiota, since our mice were treated with PTZ prior to colonization. Nonetheless, the GI-tract environment may induce cell wall stress for which *C. glabrata* requires intact *FKS1*, since *fks1Δ* mutants were highly susceptible to cell wall stresses. Moreover, cell wall alterations in such mutants may additionally induce mucosal immunity in the form of inflammation and secretion of antimicrobial peptides (45), which may further necessitate the requirement of both *FKS* during gut commensalism. Lastly, *fks1Δ* mutants may not efficiently bind to epithelial cells, potentially due to lower adhesion, which leaves more space and domination for counterparts with a higher adhesin repertoire. Future studies are required to investigate all those possibilities. The GI tract and systemic infection accordingly require niche-specific adaptation and specialized cell wall remodeling. The fact that *FKS2* activity is required for effective induction of both systemic infection and gut colonization may also increase the exposure of *FKS2* to echinocandins during treatment, which may ultimately corroborate why >2/3 of ECR *C. glabrata* isolates carry *FKS2* mutations.

Although we included an extensive array of transcriptomic, *in vitro*, *ex vivo*, and *in vivo* mouse models to understand how ECR mutations and *FKS1* and *FKS2* deletions impact the trajectory of interaction with the host, our study has multiple limitations. First, our study only included a limited spectrum of mutations, and it would be interesting to assess the fitness of other *FKS* mutations conferring ECR. Second, detailed cell wall analysis, including transmission electron microscopy and nuclear magnetic resonance, is needed to provide a high-resolution cell wall architecture and to better understand how such cell wall alterations could be linked to host recognition and which host pathogen recognition receptors play a more prominent role in recognition of WT and mutants.

## MATERIALS AND METHODS

### Growth conditions, *C. glabrata* strains, and characterization

A full loop of overnight-grown *C. glabrata* strains on YPD agar plates was inoculated in YPD broth and incubated at 37°C in a shaker incubator (150 rpm). Our study included clinical and isogenic *C. glabrata* strains (Table S3). Clinical isolates were randomly selected from a global collection of *C. glabrata* isolates with various sequence types. Isogenic ECR isolates were derived from CBS138 and were generated either by exposure to echinocandins or by CRISPR Cas9. Briefly, 10^7^ cells of CBS138 were inoculated in RPMI containing 0.125 µg/mL caspofungin and incubated at 37°C in a shaker incubator (150 rpm). The incubation was continued until the turbidity of the RPMI increased, followed by centrifugation of the pellet and transferring 10 µL of the pellet, spreading it on micafungin-containing YPD plates (0.125 µg/mL). Colonies growing on micafungin plates were subjected to HS1 and HS2 of *FKS1* and *FKS2* sequencing and antifungal susceptibility testing. *FKS1* and *FKS2* sequencing used the previously described primer and PCR and sequencing conditions (46, 47). ECR colonies harboring F659del and F659V in HS1-Fks2 were included in our extensive phenotypic and dual-RNAseq analyses. Antifungal susceptibility testing followed the Clinical Laboratory Standard Institute protocol.

ECR isolates harboring equivalent mutations in HS1-Fks1 and HS1-Fks2 were generated using a previously described methodology. Briefly, each mutation was generated by two overlapped PCR products, which were fused together by using external forward and reverse primers. Fused PCR products were subjected to sequencing to confirm the presence of the nonsynonymous HS mutation as well as the silent mutation in the PAM site. Competent *C. glabata* cells were generated using a previously described method, and transformation was performed by electroporation (48). Transformants were propagated in drug-free YPD broth for 2 h, followed by plating them on YPD plates containing 0.125 µg/mL of micafungin. Colonies growing on micafungin plates were isolated and subjected to sequencing using diagnostic primers to ensure that they contain the HS mutation. Moreover, other HS regions were also sequenced to ensure that desired mutants only contained the mutation of interest.

### Growth curves

*C. glabrata* cells grown in YPD broth overnight were washed three times with PBS and inoculated in YPD broth with an optical density (OD) of 0.1. *C. glabrata* isolates were incubated at 37°C in a Tecan Microplate Reader (Infinite 2000 pro, DKSH), and the growth of *C. glabrata* isolates was monitored for 15 h.

### Macrophage infection

THP1 macrophage derived from human acute monocyte leukemia cell line (THP1; ATCC; Manassas, VA) was used in our experiments. THP1 macrophages were grown in RPMI 1640 (Gibco, Fisher Scientific, USA) treated with 1% penicillin-streptomycin (Gibco, Fisher Scientific, USA) and 10% heat-inactivated HFBS (Gibco, Fisher Scientific, USA). Macrophage activation and attachment to 24-well plates were induced by incubating one million per well of macrophages with 100 nM phorbol 12-myristate 13-acetate (PMA, Sigma), followed by incubation in 5% CO_2_ incubator for 48 h. Subsequently, macrophages were washed with PBS and treated with RPMI 1640 at the day of infection. To expose *C. glabrata* cells, the 24-well plates containing the infected macrophages were centrifuged for 1 min at room temperature at 200 g. Macrophages were infected with the MOI of 1/1 (yeast/macrophage), and they were subjected to three times PBS washing and treated with fresh RPMI. *C. glabrata* CFU count at 3, 6, 24, and 48 h was used to determine the replication inside the macrophages. Macrophages were lysed with 1 mL of ice-cold water, and 100 µL of lysates was plated on YPD plates. IR rate was determined by dividing intracellular CFU at each time-point by CFU of the initial inoculum, and the value was presented as a percentage.

### Neutrophil isolation, infection, and flow cytometry analysis

Primary human neutrophils were collected from the blood samples of at least two healthy donors (IRB protocol #2014P002377) using the EasySep Human Neutrophil Isolation Kit (STEMCELL Technologies) in accordance with the manufacturer’s instructions. The flow cytometry strategy used a previously established protocol (32). We used an MOI of 3 yeast/1 neutrophils for experiments involving ROS and phagocytosis, whereas an MOI of 1 yeast/3 neutrophils (32). Neutrophils and *C. glabrata* cell suspensions were incubated in a CO_2_ incubator at 37°C for the desired time-points. To determine *C. glabrata* survival after interaction with neutrophils (4 h), neutrophils were lysed with PBS containing 0.02% Triton X (Millipore Sigma), followed by plating lysed neutrophils onto YPD agar plates and incubating at 37°C for 24–48 h. Similarly, the initial inoculum of each isolate was plated on YPD agar for normalization purposes, and the survival rate of *C. glabrata* isolates was determined by normalizing the CFU of lysed neutrophils to the initial inoculum of each isolate, and survival values were presented in percentage.

### Flow cytometry for intracellular replication within THP1

Macrophages were infected with the MOI of 5/1 and washed with PBS 3 h pi, followed by treatment with fresh RPMI. *C. glabrata* cells collected from macrophages at 3 and 24 h were subjected to flow cytometry, and 50,000 events were recorded for each replicate. PR was determined by collecting *C. glabrata* cells from the supernatant of the 3 h time-point. The data obtained were analyzed by FlowJo software v10.6.1 (BD Biosciences).

### Generating *fks1Δ* and *fks2Δ*

Deletant mutants of *FKS1* and *FKS2* were generated using a protocol described previously. Briefly, PCR products containing the nourseothricin-resistant gene and flanking regions complementary to outside of the *FKS1* and *FKS2* were generated using ultramer primers listed in Table S4. Electroporation-based transformation followed the previously described protocol (48). Transformants were propagated in drug-free YPD in a shaker incubator at 37°C and subsequently plated on NAT-containing YPD plates, and plates were incubated at 37°C for 48 h. Colonies growing on NAT plates were subjected to PCR using diagnostic primers, and deletants were confirmed by sequencing using the same primers.

### Macrophage cytotoxicity measurement

To measure the extent of damage of macrophages infected with *C. glabrata* isolates (MOI = 5/1), we measured the lactate dehydrogenase (LDH) using a commercial kit (Sigma) and protocol suggested previously (49). After extensive washing with PBS and adding fresh RPMI 3 h pi, macrophages were further incubated in a CO_2_ incubator for another 21 h. Subsequently, the supernatant was collected and subjected to an LDH determination experiment. The values obtained for each replicate were subtracted from the untreated background and divided by the value of the high control (macrophages treated with 0.25% Triton 100-X for 3 min), and the corrected normalized values were presented as a percentage.

### Cell wall analysis

Overnight-grown *C. glabrata* cells (in YPD) were extensively washed with PBS, and following adjusting the cell density to OD_600_ of 0.4, their cell wall components were analyzed using a flow cytometry-based technique described previously (31, 32). Concanavalin A AF-647, wheat-germ agglutinin-FITC, and anti-β-glucan primary antibody (Biosupplies) were used to detect mannan, chitin, and β-glucan, respectively. The concentrations, incubation temperatures, and times are detailed in previous studies (31, 32).

### Basal ROS determination

Overnight-grown *C. glabrata* cells in YPD were extensively washed with and resuspended in PBS. PBS solution containing 1/1,000 DHR123 (Thermo Fisher) was added to each replicate (50,000,000 CFU), followed by incubation at 37°C for 30 min. Upon incubation times, the ROS level was assessed after centrifugation of samples and resuspending in PBS. Control strain not treated with DHR123 was used to gate the area of interest, and MFI for each sample was recorded.

### Growth curve analysis

Overnight-grown *C. glabrata* cells (in YPD) were extensively washed with PBS and reached an optical density 600 of 0.2 in a desired media, followed by 24-h incubation at 37°C (unless stated otherwise) in a microplate reader (SpectraMax i3x multimode detection platform, VWR).

### Catalase-level analysis

*C. glabrata* isolates grown overnight in YPD were subjected to washing with PBS and resuspended in PBS. 2,000,000 CFU of each isolate (25 µL) along with catalase standards were added to 96-well plates, mixed reaction buffer containing either 40 µM or 10 mM (25 µL), and incubated at room temperature for 30 and 360 min, respectively. Following the addition of HRP and Amplex Red to each sample (50 µL), samples were incubated at 37°C for 15 min, followed by recording fluorescence (excitation of 550 nm and emission of 590 nm) using a microplate reader (SpectraMax i3x multimode detection platform, VWR). Catalase level was determined based on standards as suggested by the manufacturer (Amplex Red, Thermofisher).

### DNA extraction, library preparation, and Illumina sequencing sample quality assessment

The genomic DNAs were prepared using the ZymoResearch kit (Irvine, California, USA). DNA library preparation, sequencing, and initial bioinformatics analysis were conducted at Azenta Life Sciences (South Plainfield, NJ, USA). Genomic DNA was quantified using the Qubit 2.0 Fluorometer (ThermoFisher Scientific, Waltham, MA, USA). NEBNext UltraTM II DNA Library Prep Kit for Illumina was used for library preparation, following the manufacturer’s recommendations. Briefly, the genomic DNA was fragmented by acoustic shearing with a Covaris S220 instrument. Fragmented DNA was cleaned and end-repaired. Adapters were ligated after adenylation of the 3′ ends, followed by enrichment by limited-cycle PCR. DNA libraries were quantified using Qubit 2.0 Fluorometer. The DNA libraries were also quantified by real-time PCR (Applied Biosystems, Carlsbad, CA, USA). The sequencing libraries were clustered onto a 25B flow cell on the Illumina NovaSeq X Plus instrument according to the manufacturer’s instructions. The samples were sequenced using a 2 × 150 paired-end (PE) configuration, targeting ~1 Gb/sample. The control software conducted image analysis and base calling. Raw sequence data (.bcl files) generated by the sequencer were converted into fastq files and de-multiplexed using Illumina’s bcl2fastq 2.20 software. One mismatch was allowed for index sequence identification.

### Whole-genome sequencing analysis

Whole-genome sequence data were analyzed exactly as described previously (50), with the only difference that the reference strain in this study was CBS138.

### Whole-genome sequencing results

Fks2^F659del^ showed extreme fitness defects under various *in vitro* and *ex vivo* conditions, and unlike the rest of the iECR mutants generated by CRISPR-Cas9, the Fks2^F659del^ and Fks2^F659V^ mutants evolved through caspofungin exposure. Therefore, we speculated that these mutants may carry additional genomic mutations, some of which may underlie the sick phenotype of Fks2^F659del^ mutants. Therefore, we performed whole-genome sequencing for all those iECR isolates, including Fks1^S629P^ (*n* = 1), Fks1^R631G^ (*n* = 2), Fks2^S663P^ (*n* = 2), Fks2^R665G^ (*n* = 1), Fks2^F659V^ (*n* = 1), and Fks2^F659del^ (*n* = 2). Surprisingly, Fks2^F659V^ and Fks2^F659del^ did not carry deleterious mutations in genes implicated in key cellular functions, such as oxidative stress response, which further emphasizes that the severe fitness defect of Fks2^F659del^ is potentially driven by the Fks2 mutation (Table S1). One of the Fks2^S663P^ carried nonsynonymous mutations in YHB1 and NUP100, and the other one in POL12, and both Fks1^R631G^ harbored non-synonymous mutations in KAR2 (Table S1). Nonetheless, it is unlikely that those mutations confer any appreciable impact on fitness as those ECR mutants did not suffer a fitness defect, especially during interaction with phagocytes. Collectively, our whole-genome sequencing analysis indicated that the severe fitness defect of the Fks2^F659del^ may not be explained by additional genomic changes deleteriously impacting the open reading frame of genes involved in key cellular functions. The whole-genome sequence data were deposited into the public repository and assigned accession number PRJNA1372781.

### RNA extraction

Macrophages infected with the MOI of 5/1 were extensively washed 3 h pi, and fresh RPMI was added to wells to be further incubated at 37°C. After extensive PBS wash at each step, macrophages were subjected to a manual RNA extraction protocol described elsewhere (49), followed by RNase-free-DNase and further purification using the RNeasy kit (QIAGEN) per the manufacturer’s instructions. The integrity and quantity of RNA samples were confirmed by running RNA samples in 1.5% agarose gel and using NanoDrop (ThermoFisher), respectively.

### Library preparation and sequencing

Library preparation and RNAseq were carried out by Azenta Life Sciences in the exact same manner as described previously (22).

### Dual RNAseq data analysis

Raw sequencing data were subjected to quality control using FastQC v. 0.11.8 (https://www.bioinformatics.babraham.ac.uk/projects/fastqc/) and MultiQC v. 1.1 (51). Trimming was conducted with Trimmomatic v. 0.36 (52), using the parameters <TruSeq adapters: 2:30:10 LEADING:3 TRAILING:3 SLIDINGWINDOW:4:3 MINLEN:50>.

Read mapping and quantification were performed via STAR v. 2.7.10a (53), a splice junction-sensitive read mapper, with default parameters. Reads from samples containing only human or fungal RNA were mapped to their respective reference genomes, whereas those from mixed samples were aligned to the combined reference genomes. For human data, we employed the T2T CHM13v2.0 telomere-to-telomere genome assembly (54) and annotations from NCBI (accessed 12 May 2022). The human mitochondrial genome from GRCh38 was obtained from the Ensembl database (accessed 12 May 2022) (55). Reference genomes and annotations for *C. glabrata* CBS138 were retrieved from the Candida Genome Database (CGD, accessed 12 May 2022) (56). Potential read cross-mapping (i.e., reads that map equally well to both human and fungal genomes) was assessed using crossmapper v. 1.1.1 (57). Differential gene expression analysis was conducted with DESeq2 v. 1.26.0 (58). Genes with |log2 fold change (L2FC)| > 1 and adjusted *P*-value (*P*adj) < 0.01 were considered as differentially expressed. Gene ontology (GO) term enrichment (Biological Process category) was performed using ClusterProfiler v. 3.14.3 (59). GO term association tables for *C. glabrata* were obtained from CGD (accessed 12 May 2022), while annotations for human data were sourced from the Genome wide annotation for Human (org.Hs.eg.db) database v. 3.10.0. The raw RNA sequencing data are accessible via accession number PRJNA1230055.

### GI-tract colonization mice model

The protocols concerning the animal experiments (GI tract and systemic infection) were reviewed by the pertinent Institutional Animal Care and Use Committee (IACUC) and comply with the Public Health Service and National Institute of Health Policy of Humane Care and Use of Laboratory Animal Guide.

Six-week-old CF-1 female mice (Charles River Laboratory) were used for GI-tract colonization using a previously described protocol (42). To eradicate the mice’s GI-tract commensal bacteria, mice were treated with piperacillin-tazobactam (PTZ), starting 2 days before the colonization and continuing until the last day of the experiment. *C. glabrata* cell suspensions containing the mixture of two isolates (100 µL of 1.5 × 10^8^ mixtures) were transferred via oral gavage. Fecal samples collected on days 1, 3, 5, and 7 post-colonization were plated on YPD plates containing PTZ and incubated at 37°C for 48 h. The proportion of GFP-expressing and non-fluorescent *C. glabrata* colonies was determined by subjecting plates to Typhoon (dark colonies = GFP and light colonies = non-fluorescent). The proportion of each isolate was determined by dividing the CFU of a given isolate by the total CFU, and the proportion was presented as a percentage.

### Mouse systemic infections

Six-week-old CD-1 female mice were used for systemic infection using a previously described protocol (42). Mice were immunosuppressed prior to infection by administration of 150 mg/kg cyclophosphamide 4 days before infection, followed by administration of 100 mg/kg once every 3 days until the last day of the experiment. Mice were infected via the rhino-orbital route using 50 µL of cell suspensions (5 × 10^7^) containing the mixture of two isolates. Each competition included 12 mice and 4 mice that were euthanized and sacrificed at each designated timepoints (days 1, 4, and 7). Subsequently, the harvested kidney and spleen were homogenized extensively and plated on YPD agar plates, followed by incubation at 37°C for 48 h. The proportion of GFP-expressing and non-fluorescent *C. glabrata* colonies was determined by subjecting plates to Typhoon (dark colonies = GFP and light colonies = non-fluorescent). The proportion of each isolate was determined by dividing the CFU of a given isolate by the total CFU, and the proportion was presented as a percentage.

### Statistical analysis

We used SPSS software (v.24 for Windows; SPSS, Inc., Chicago, IL, USA) for our statistical analysis, and *P*-values ≤0.05 were considered to indicate statistical significance. Data distribution was determined using the Shapiro-Wilk test, and statistical significance of nonparametric data was determined using the Wilcoxon test.

## SUPPLEMENTAL MATERIAL

Fig. S1 aac.01801-25-s0001.tif The RNAseq data of intracellular *C. glabrata*.

Fig. S2 aac.01801-25-s0002.tif The RNAseq data of macrophages infected with *C. glabrata*.

Table S1 aac.01801-25-s0003.xlsx Summary of the whole-genome sequence data.

Table S2 aac.01801-25-s0004.xlsx The RNAseq data of *C. glabrata* during interaction with THP1 macrophages.

Table S3 aac.01801-25-s0005.xlsx List of clinical and isogenic *C. glabrata* isolates used in the current study and their sequnce type, antifunagl susceptibility profiles, and FKS and PDR1 mutations.

Table S4 aac.01801-25-s0006.xlsx List of oligos, their functions, and amplification programs.

ASM does not own the copyrights to Supplemental Material that may be linked to, or accessed through, an article. The authors have granted ASM a non-exclusive, world-wide license to publish the Supplemental Material files. Please contact the corresponding author directly for reuse.
